# Experimental Researches on the Post-Mortem Contractility of the Muscles, &c.

**Published:** 1847-04-01

**Authors:** 


### Experimental Researches on the Post-mortem Contractility of the Muscles, with Observations on the Reflex Theory. By *Bennet Dowler*, M.D. New York, 1846.

It happens, from time to time, that some writer favours the world with speculations directly opposed to what are, by common consent, received as established laws and principles in science. On such productions people usually, and with good reason, look with some degree of suspicion; for, in order to make room for their reception, the labours of the most splended minds must first of all be set aside. The brochure before us belongs to the category just indicated. The author, not satisfied with criticising what are, to him, the errors of Haller, sweeps away the ground-work on which Bell based his splendid discoveries; and, having accomplished this feat to his own satisfaction, naturally enough asks whether, after all, there be any discovery in the doctrines of our distinguished countryman.

After such achievements, it is a small matter that our author demolishes without remorse, nay, with some indications of inward satisfaction, the whole theory of the reflex action, treating Dr. Marshall Hall with much less ceremony than is extended to his illustrious predecessor. Our readers will have formed great expectations of a work having such lofty objects; and may, if former experience has not cooled down such anticipations, be anxious to learn the character of these "Researches." For the sake of truth, and for the vindication of all sound physiology, we must place a very low value upon Dr. Dowler's investigations, whether regarded in the light in which it is wished that they should be viewed, namely, as original researches, or as invalidating the fundamental laws of innervation. We have no wish, however, to affirm, that these observations are devoid of interest; nor that, if published as illustrative of a somewhat obscure class of phenomena connected with muscular action, they would have been uninstructive; but, considered as the lever by which the magnificent superstructure of modern neurology is to be overturned, we hold them to be most vain and futile. On looking over the arguments and statements of Dr. Dowler, it becomes, moreover, immediately apparent, that his acquaintance with the anatomy and physiology of the present day is most defective; and one consequence is, that he brings forward as a novelty what is familiarly known to all careful observers on this side the Atlantic, namely, that muscular contractions, occasionally of a powerful character, so that the limbs are moved, may take place shortly after death. Another result of his imperfect knowledge is, that the author is ignorant of a large body of evidence, which has brought conviction to the professional mind of Europe, that the doctrine of the spinal action is a great truth, henceforth to be ranked as one of the fundamental principles of physiological science. Such indeed is the agreement upon this subject, that it would be useless to waste our own space and the time of our readers, by any lengthened refutation of the crude and fallacious reasoning with which we are informed our American brethren were first favoured in the New York Journal of Medicine. A few extracts will exhibit the physiological acumen and qualifications of a writer who has the ambitious aspirations we have just noticed.

"The reflex school maintains, not only that the integrity of the spinal cord is indispensable to transmission, but that the division of the anterior roots is a complete barrier to muscular motion. This doctrine is not based on the healthy, living body. It is not, with a few obscure and unimportant exceptions, deduced from morbid conditions, but from the last agony, and more than all, from the recently dead state of the inferior animals—a kind of proof by no means satisfactory.

"It should never be forgotten that experiments on the inferior animals, as frogs and turtles, are inconclusive in establishing the complicated physiology of man." P. 8.

It is difficult to conceive, with the evidence possessed upon the points here referred to, how this passage could have been penned: the only possible explanation is the one above suggested. What, it may be asked, are the phenomena displayed in the anencephalous infant that survives its birth? It breathes, it cries, it sucks, it discharges the excreta of the body. How, we would ask of the author, are these complex, associated muscular movements performed? Do they involve *any* nervous agency?—if so, what is the part implicated? Brain there is none; and we may presume that, even Dr. Dowler would not attribute either to the nerves or to the great sympathetic, the power of originating and combining in functional action, muscles so numerous and remote as those engaged in the functions named. What other conclusion remains, but that the spinal cord is the necessary and active centre. As to the objections raised against vivisections, they do not apply to the case before us. If it can be shown that, in a decapitated turtle or frog, or in a rabbit with a portion of the spinal cord removed, impressions made on the skin produce definite, and as it has been clearly proved, functional muscular actions; if it can be shown that the same results are, proportionally to the parts excited, induced by irritating the posterior roots of the nerves, whilst they are arrested by the division of the anterior roots, and are totally prevented by the destruction of the cord; admitting these phenomena, can any one acquainted with the unity of the organic laws, that one great truth which has been evolved from the profound researches of Cuvier, of Owen, and of every successful cultivator of this branch of science, can any one, we ask, entertain a doubt that, the conditions being the same, the consequences would also be the same in man, with a spinal centre constructed upon essentially similar principles to that of reptiles and mammals? If such kind of evidence be rejected, physiology must return to its very infancy; for, with a few exceptions, little or nothing can be learnt, strange as it may sound to some ears, of human physiology from observations exclusively restricted to man. The difficulty of drawing safe deductions from experiments made on animals, relates to the phenomena of sensation, not of motion, which, as a general rule, can be certainly and successfully interpreted.

In further illustration of his argument the author invokes the supposed fact that "an earth-worm may be cut into several pieces, and that each portion becomes a perfect animal." No one acquainted with the structure of this annelide and with the laws of development, could imagine such a departure from the principles of formation; but, for the information of Dr. Dowler, we may state that, by numerous experiments made some years ago, we ascertained that no portion of the earth-worm severed from the head, however large, survived beyond a limited period, dependent upon the length of the segment: the part so detached dies ring by ring.

It would, however, be useless to dwell longer on the errors with which the work abounds, in reference to the received opinions of physiologists; we therefore proceed to notice briefly the author's own researches, into the mysteries of which we are thus somewhat facetiously initiated. "During an attempt to produce contusions on the recently dead body, I happened to select a spot over the middle of the biceps; a blow there (the arm having been extended on the floor) caused the subject to slap his hand against his face with much force. I was almost as much surprised as was a black man that I hired to aid me in a private dissection in St. John the Baptist street, in 1842, of the body of a gentleman of much travel, who died of remittent fever. Previous to this dissection, which was two hours after death, I struck the arm with the inferior edge of my extended hand. The subject contracted his arm, carrying his hand to his breast. My aid looked to the door, which had been closed beforehand, and begged to be let out without delay."

This post-mortem muscular contractility Dr. Dowler regards not only as a thing, till seen by him, unknown; but likewise as an important point of departure in the analysis of some of the most interesting problems in physiology. Many other similar instances, out of a very large number, are related; being principally cases of death from yellow fever, that occurred at New Orleans. One or two of these we extract:—

"Mr. S., aged 45. Dead two hours; legs becoming rigid; struck the flexors of the arm with the inferior edge of my hand; the cadaver raised his arm with a regular slow movement, placing his hand upon his breast: as soon as the muscles relaxed, he carried his arm back, extending the same. The experiment was repeated three or four times, when the arm fell back exhausted. The blows were now made with a piece of wood. The biceps gathered up into a lump, at the place where the blow was given, but failed to move the fore-arm." P. 19.

The following and other cases show that calorification may take place after death:—

"J. K., a Philadelphian, aged 25. In fifteen minutes after death presented the contractile phenomena in its most intense form, but which declined wholly in one hour, the body being everywhere flexible. In half an hour after rigidity set in. This body, which before death had been remarkably cold, had a temperature after death as high as 109°, and which did not refrigerate below 104° in three hours after."

In reference to this development of heat, the author observes, "the continuance of, or rather the degree in which post-mortem heat is evolved bears no proportion, I repeat, to the intensity of post-mortem contraction. The great heat developed in the dead body, I have endeavoured to illustrate in the medical journals of our country, and will not, therefore, dwell upon that subject at present. I find, however, on examination of the original papers not yet published in detail, that for the most part, when the heat had declined the contractility was exhausted, but that the presence of great heat, ranging as high as 113°, did not by any means imply the presence of contractility, nor the absence of rigidity. Authors seem not to have been aware of the augmentation of animal heat after death; some have, it is true, noticed an increase of heat after death from cholera, compared with the extreme coldness of the surface during the last hours of life: but has any one hinted that this post-mortem heat ever rose as high as even the healthy standard, to say nothing of 14° or 15° beyond that?" P. 23.

The cause of the contractions above described, and similar instances occurred in this country during the prevalence of cholera, is doubtless the *rigor mortis*, and is, therefore, independent of the nervous centres. Such is the conclusion of the author; but it is also the opinion of physiologists generally, in the present day. Mr. Bowman was the first writer who distinctly showed by microscopic observation, that the individual muscular fibres contracted independently of the presence of nerves; and we have here the clue to the more extensive, but essentially the same phenomena related by Dr. Dowler, which, therefore, can have no bearing upon the question of the spinal action. If the republication of these views be the result of any peculiar importance attached to them on the other side of the Atlantic, we fear that modern physiology has not penetrated very deeply into the American professional mind; with this remark we must dismiss these most crude "Experimental Researches."

